# Impact of Protein Content on the Antioxidants, Anti-Inflammatory Properties and Glycemic Index of Wheat and Wheat Bran

**DOI:** 10.3390/foods11142049

**Published:** 2022-07-11

**Authors:** Ivan Jesus Jimenez-Pulido, Rico Daniel, Jara Perez, Cristina Martínez-Villaluenga, Daniel De Luis, Ana Belén Martín Diana

**Affiliations:** 1 Agrarian Technological Institute of Castilla and Leon (ITACyL), Ctra. Burgos Km 119, Finca Zamadueñas, 47071 Valladolid, Spain; jimpuliv@itacyl.es (I.J.J.-P.); mardiaan@itacyl.es (A.B.M.D.); 2 Institute of Food Science, Technology and Nutrition (ICTAN-CSIC), Department of Metabolism and Nutrition, Juan de la Cierva, 3, 28006 Madrid, Spain; jara.perez@ictan.csic.es; 3 Institute of Food Science, Technology and Nutrition (ICTAN-CSIC), Department of Technologycal Process and Biotechnology, Juan de la Cierva, 3, 28006 Madrid, Spain; c.m.villaluenga@csic.es; 4 Endocrinology and Nutrition Research Centre, Service of Endocrinology and Nutrition, Universitary Clinic Hospital of Valladolid, University of Valladolid, Av. Ramón y Cajal, 3, 47003 Valladolid, Spain; dluisro@saludcastillayleon.es

**Keywords:** valorization, wheat bran, antioxidant activity, glycemic index, anti-inflammatory activity

## Abstract

Conventional wheat milling generates important volumes of wheat bran (WB), which is a concentrated source of polyphenols and insoluble fiber. In terms of health benefits and based on epidemiological and experimental evidence, these compounds contribute to reducing the risk of certain chronic pathologies. Protein concentration is the main quality factor conditioning wheat use in the agroindustry. When turning waste into feasible resources, it is essential to evaluate the variability of the raw material. The aim of this study was the evaluation of the impact of protein content in the valorization of WB based on its antioxidants, anti-inflammatory properties and glycemic index (GI). A significantly (*p* ≤ 0.05) lower content of phenolic compounds was found in the whole grain (WG) fractions, both free (FP) and bound (BP), as compared to the WB phenolic fractions, differences that ranged from 4- to 6-fold (538 to 561 mg GAE 100 g^−1^ in WG vs. 1027 to 1236 in WB mg GAE 100 g^−1^ in FP and 2245 to 2378 vs. 6344 to 7232 mg GAE 100 g^−1^ in BP). A significant (*p* ≤ 0.05) effect of the protein content on the resulting phenolic content and antioxidant capacity was observed, especially in WG, but also in WB, although in the latter a significant (*p* ≤ 0.05) negative correlation was observed, and increasing the protein content resulted in decreasing total phenolic content, antioxidants, and ferric-reducing capacities, probably due to their different types of proteins. The highest protein content in WB produced a significant (*p* ≤ 0.05) reduction in GI value, probably due to the role of protein structure in protecting starch from gelatinization, along with phytic acid, which may bind to proteins closely associated to starch and chelate calcium ions, required for α-amylase activity. A significant (*p* ≤ 0.05) effect of the protein content on the GI was also found, which may be explained by the structural effect of the proteins associated with starch, reducing the GI (21.64). The results obtained show the importance of segregation of WB in valorization strategies in order to increase the efficiency of the processes.

## 1. Introduction

The concept of a circular economy (CE) has received growing interest worldwide in the recent decade. Indeed, the European Commission (EU) adopted a new strategy, known as the new circular economy action plan, in 2020, and this being one of the most important plans of the European Green Deal. The objective of this strategy is to reduce the pressure on natural resources and contribute to a sustainable growth, and create new opportunities for the industry, CE being a prerequisite to achieve in the EU’s 2050 climate neutrality target [1]. CE is based on three main pillars: firstly, the environmental benefits; secondly, cost savings from reduced resource use; and thirdly, the generation of new market opportunities. In this sense, the agroindustry faces new challenges and opportunities. The implementation of new action strategies is important overall in the agriculture sector, which generates an important volume of by-products.

Wheat (*Triticum aestivum* L.) is the second most consumed grain crop in the world, with special relevance in the Mediterranean region [2], and the third most important crop in terms of global production, after maize and rice [3]. It is one of the most important staple foods since it meets most of the protein requirements, and in 2019/2020 the global demand for wheat reached 762.4 million tons [4].

Most wheat grain (WG) are milled into different types of refined flours, producing a by-product known as wheat bran (WB), which represents about 15% of WG weight [5]. WB is the outer layer of the wheat kernel, which is separated from the endosperm and germ during the milling process. It is composed of the outer grain layers (cuticle, pericarp and seed coat), with small quantities of endosperm from the wheat kernel [6,7,8,9,10]. WB contains protein (13–18%), fat (3–5%) and carbohydrates (50–60%), of which 70–90% is dietary fiber (DB) [11]. Other brans, especially oat bran, are quite popular in human nutrition, mainly associated with their beta-glucan content, while wheat bran is mainly used for animal feed [12].

There are different research studies indicating that the consumption of foods and wholegrains with high content in dietary fiber have important benefits in gastrointestinal disorders, such as constipation, hemorrhoids and diverticulitis [13]. Moreover, the intake of wheat bran and other foods high in dietary fiber contribute to satiety, which has important benefits, such as maintaining a healthy status and weight [14]. Reynolds et al. [15] also reported the importance of dietary fiber in the control of cardiovascular diseases thorough the application of metanalysis studies.

In addition, WB is a source of micronutrients, such as vitamins, minerals and other botanical compounds, such as alkylresorcinols, flavonoids, carotenoids, lignans, sterols and phenolic acids [6,7,12]. Phenolic acids and flavonoids appear in free, soluble conjugated and insoluble bound forms. Wheat phenolic acids include the most abundant compounds ferulic, vanillic, syringic, sinapic, caffeic and *p*-coumaric acids, which have important antioxidants properties [16]. These antioxidants, such as phenolic compounds, have been shown to regulate proinflammatory signals and angiogenesis, thus participating in the immune system [17]. Phenolic compounds in wheat have been shown to activate monocyte adhesion to the endothelium and to reduce the expression of proinflammatory cytokines [18]. Phenolic acids are the most abundant phenolic compounds in wheat and appear in free, conjugated and bound forms, the latter being the most abundant [19]. The antioxidant capacity of wheat phenolic compounds has been previously reported by different authors [8,10,20,21]. This has been described as LDL oxidation inhibition, oxidative stress reduction, blood pressure modulation, plasma cholesterol and triglycerides reduction, as well as type 2 diabetes development, cardiovascular diseases (CVD) and certain cancer types prevention [19].

Furthermore, technological strategies focused on improving the release of food containing bioactive compounds in the organism [22] can help to increase the valorization of many bioactive-rich by-products and help in the implementation of a circular-based economy [8].

Previously, a strategy to promote the valorization and development of nutraceutical ingredients from WB, such as mechanical treatments, for particle size reduction, combined with hydrothermal treatments, has been proposed [8,23]. This strategy explored the use of enzymatic treatments to improve the extraction and solubilization of bioactive compounds from WB [23]. In particular, sequential and enzymatic treatments of WB with Ultraflo XL at optimal conditions (47 °C, pH 4.4 for 21 h) maximized the free ferulic acid content and its capacity to scavenge oxygen radicals, chelate transition metals and inhibit secretion of cytokines in a cellular model of inflammation [23]. However, waste valorization strategies are also conditioned by raw material variability, an aspect that is most of the time overseen. Protein concentration is the main quality factor conditioning wheat end-use in the agroindustry [24,25,26]. The bran that the wheat-milling industry produces as a by-product is highly variable. One of the main factors that determine this variability is the protein content of the grain, which affects the antioxidant properties of grain bran, with a significant impact on total phenolic content and antioxidant properties [26,27,28,29].

Since the milling industry produces WB with a high variety of wheat sources, it is necessary to evaluate the effect of the grain protein content on the bran produced; therefore, the objective of this study was to evaluate the influence of WG protein content on the grain and bran antioxidants, anti-inflammatory properties and glycemic index, in order to better assess this variability of the obtained bioactive properties and to improve wheat bran valorization strategies, for the development of nutraceutical ingredients.

## 2. Materials and Methods

### 2.1. Chemicals

Folin–Ciocalteu (FC) reagent, gallic acid (GA), 6-hydroxy-2,5,7,8-tetramethyl-2-carboxylic acid (Trolox), 2,20-diazobis-(2-aminodinopropane)-dihydrochloride (AAPH), fluorescein, 2,20-azinobis 3-ethylbenzothiazoline-6-sulfonic acid (ABTS•+), and 2,2-diphenyl-1-picrylhydrazyl (DPPH), 2,4,6-tripyridyl-triazine (TPTZ), iron(III) chloride hexahydrate (FeCl_3_∙6H_2_O) and iron(II) sulfate heptahydrate (FeSO_4_∙7H_2_O), apigenin, kaempferol, (-)-epicatechin, ferulic acid, gallic acid, p-coumaric acid, sinapic acid, avenanthramide C and secoisolariciresinol were obtained from Sigma-Aldrich, Co. (St. Louis, MO, USA). Amyloglucosidase (EC 3.2.1.3) and glucose oxidase-peroxidase (GOPOD) were provided by Megazyme (Wicklow, Ireland). Sodium acetate, chlorhydric acid and glacial acetic acid were obtained from PanReac AppliChem (ITW Reagents, Darmstadt, Germany). Solvents were HPLC grade (Sigma Aldrich Co., Madrid, Spain, and Merck KGaA, Darmstadt, Germany).

### 2.2. Materials

Three different wheat (*Triticum aestivum* L.) grains and their corresponding brans were used in this study and kindly supplied by the milling company Emilio Esteban, S.A. (Valladolid, Spain). The wheat was harvested in Valladolid in 2020. The three wheat samples had three different protein concentrations, and referred to as high protein (WG HP), medium protein (WG MP) and low protein (WG LP) samples. The brans were produced using a dry-milling process, which involves the separation of bran from the endosperm. WB and WG were milled with a Laboratory Mill 3100 (Perten Instruments, Hägersten, Sweden) to reduce the particle size until more than 95% of the bran particles passed through a 500-μm sieve. Samples were stored in plastic bags under vacuum until analysis.

### 2.3. Proximate Composition

Three grams of powdered sample (grain or bran) were dried at 105 °C for 3 h to determine the moisture content. The total protein content was measured by the Dumas method, 990.03 [30], in an elemental analyzer (LECO Corp., St. Joseph, MI, USA). To convert nitrogen into protein values a conversion factor of 6.25 was used. A Soxtec extracting unit (AOAC 2005, method 2003.05) [30] was used to determine the total fat content with petroleum ether extraction (40–60 °C) for 4 h.

For the ash content, samples were incinerated in a muffle furnace at 550 °C for 5 h (AOAC 2005, method 923.03) [30]. Carbohydrates were estimated by difference. Total dietary fiber (TDF) content was determined using the TDF100A-1KT assay kit provided by Sigma (St. Louis, MO, USA), in accordance with the manufacturer’s instructions, based on AOAC method 985.29 [30]. Phytic acid (PA) and total starch content (TSC) were determined using the K-PHYT and K-TSTA-100A assay kits (Megazyme, Wicklow, Ireland), respectively. All results were corrected for moisture content and expressed as g 100 g^−1^ of dry matter (d.m.). All analyses were performed in duplicate.

### 2.4. Phenolic Extracts Preparation

Free and bound phenolic compounds were extracted from different WB and WG samples following the procedure described by Dinelli et al. [31].

#### 2.4.1. Release of Free Phenolic Compounds (FP)

One gram of each WB was dissolved in 20 mL of chilled EtOH/H_2_O (80:20, *v*/*v*) for 10 min at room temperature using magnetic agitation. After centrifugation (25 °C, 2500× *g*, 10 min) the supernatant was collected, and the extraction was repeated twice. All supernatants were pooled and evaporated to dryness on a rotary evaporator (Rotavapor R-210, Buchi, Switzerland) under vacuum at low temperature (45 °C). The dried extracts were resuspended in 10 mL of MeOH/H_2_O (80:20, *v*/*v*) and filtered through a nylon filter (0.22 μm) and stored at −80 °C until analysis.

#### 2.4.2. Release of Bound Phenolic Compounds (BP)

The pellet obtained after centrifugation during extraction of free phenolic compounds (2.4.1.) was subjected to alkaline and acid hydrolysis to recover the bound phenolic compounds. A total of 12 mL of distilled water and 5 mL of 10 M NaOH were added to the residue and stirred overnight at room temperature using a magnetic stirrer. The pH of the solution was adjusted to pH 2, and the released phenolic compounds were extracted three times with 15 mL of ethyl acetate by manual shaking and centrifugation (25 °C, 2500× *g*, 10 min). The ethyl acetate layers were polled and refrigerated.

After alkaline hydrolysis, acid hydrolysis was carried out by adding 2.5 mL of concentrated HCl and incubated in a water bath at 85 °C for 30 min. The sample was cooled down and phenolic compounds were extracted with ethyl acetate in the same way as described above. Fractions obtained from alkaline and acid hydrolysis were mixed and evaporated to dryness with a rotary evaporator (40 °C). The extracts were reconstituted with 10 mL of MeOH and filtered through a nylon filter (0.22 μm) and stored at −80 °C until analysis.

### 2.5. Determination of Total Phenolics Compounds (TP)

Folin–Ciocalteu phenol reagent was used to measure the TP content in the fractions of free and bound phenolic compounds, according to Slinkard and Singleton [32]. The absorbance was measured at 765 nm using a microplate reader (Fluostar Omega, BMG, Ortenberg, Germany). Gallic acid was used as standard (500–100 μM). Results were expressed as mg gallic acid equivalents (GAE) 100 g^−1^ d.m. All analyses were performed in duplicate.

### 2.6. Characterization of Phenolic Compounds by HPLC-ESI-QTOF-MS

WG and WB, free and bound polyphenol fractions were analyzed by HPLC-ESI-QTOF-MS. A HPLC apparatus (Agilent 1200, Agilent Technologies, Santa Clara, CA, USA) with DAD (Agilent G1315B) and a QTOF mass analyzer (Agilent G6530A) with an atmospheric pressure electrospray ionization (ESI) was used for separation. The column used was 250 mm × 2 mm i.d., 5 μm, Luna C_18_ (Phenomenex, Torrance, CA, USA) at 25 °C. Gradient elution was performed with 0.1% aqueous formic acid (solvent A) and 0.1% formic acid in acetonitrile (solvent B). The gradient applied at a flow rate of 0.4 mL/min was as follows: 8% B, 0 min; 23% B, 10 min; 50% B, 15 min; 50% B, 20 min; 100% B, 23 min; followed by a re-equilibration step. The volume injected was 2 μL. Data were acquired with the negative ion mode with a mass range of 100–1200 Da, a source temperature of 325 °C and a gas flow of 10 L/h. Compound identification was verified with retention times of commercial standards when available. Otherwise, the molecular formulas proposed by the MassHunter Workstation software version 4.0 for the different signals obtained in the MS experiments were compared with those of phenolic compounds previously reported in wheat and other cereals, and accepted with a maximum error of 10 ppm. Additionally, the auto MS/MS acquisition mode was applied for the MS/MS experiments; the fragmentation patterns reported for phenolic compounds were used to compare to the main fragments obtained.

Quantification of the phenolic compounds was performed using calibration curves of authentic standards (apigenin, (-)-epicatechin, kaempferol, ferulic acid, gallic acid, p-coumaric acid, sinapic acid at a concentration range between 0.1 and 25 μg mL^−1^, showing good linearity (R^2^ > 0.99). Results were expressed as the mean ± standard deviation of two independent replicates in mg 100 g^−1^ d.m.

### 2.7. Total Antioxidant Capacity (TAC)

The TAC was determined in the extracts by DPPH radical scavenging activity, ABTS•+^•+^ radical cation scavenging activity, oxygen radical absorbance capacity (ORAC) and ferric reducing antioxidant power (FRAP). All analyses were performed in duplicate.

#### 2.7.1. DPPH Radical Scavenging Activity

The DPPH assay was carried out according to the procedure described by Brand-Williams et al. [33], with modifications. A volume of 25 μL of sample was mixed with 100 μL of milliQ water and 125 μL of DPPH working solution (120 μM in pure methanol) in a 96-well microplate. Absorbance was measured at 515 nm for 30 min with a microplate reader (Spectrostar Omega, BMG, Ortenberg, Germany). A Trolox curve was used as a standard (7.5–210 μM). Results were expressed as μmol of Trolox Equivalents (TE) 100 g^−1^ sample (d.m.).

#### 2.7.2. ABTS•+^•+^ Radical Cation Scavenging Activity

The ABTS•+^•+^ assay was carried out according to Re et al. [34], modified by Martin-Diana et al. [35]. A total of 20 μL of the sample was mixed with 200 μL of ABTS•+^•+^ working solution in a 96-well microplate. Absorbance was measured at 730 nm for 60 min with a microplate reader (Spectrostar Omega, BMG, Ortenberg, Germany). A Trolox curve was prepared as a standard (7.5–210 μM). Results were expressed as μmol TE 100 g^−1^ sample (d.m.).

#### 2.7.3. Oxygen Radical Absorbance Capacity (ORAC)

The ORAC assay was performed according to the method reported by Ou et al. [36], with slight modifications. Samples and Trolox standard curve (7.5–210 μM) were diluted with phosphate buffer (10 mM, pH 7.4). A volume of 25 μL of Trolox standard, sample, and phosphate buffer as blank and a volume of 150 μL of fluorescein were added to a black 96-well microplate. They were incubated at 37 °C for 3 min before adding the AAPH solution to initiate the oxidation reaction. Fluorescence was monitored for 100 min with a microplate reader (Fluostar Omega, BMG, Ortenberg, Germany) using 485 nm excitation and 520 nm emission filters. Results were calculated by plotting the areas under the fluorescein decay curves, between the blank and sample, and expressed as μmol TE 100 g^−1^ sample (d.m.).

#### 2.7.4. Ferric Reducing Antioxidant Power (FRAP)

The FRAP assay was performed following the procedure reported by Benzie and Strain [37], with slight modifications. A 300 mM acetate buffer, pH 3.6 (mixing a solutions of 300 mM sodium acetate and 300 mM glacial acetic acid until pH 3.6), a 10 mM TPTZ (2,4,6-tripyridyl-triazine) solution in 40 mM HCl, and a 20 mM FeCl_3_∙6H_2_O solution were prepared. The FRAP working solution was prepared by mixing the acetate buffer, TPTZ solution and FeCl_3_∙6H_2_O solution in a 10:1:1 ratio of volumes. A curve of FeSO_4_∙7H_2_O was prepared as the standard (400–3000 μM). A total of 20 μL of the sample, standard or water as blank, was mixed with 1.9 mL of the FRAP working solution in Eppendorf tubes. They were incubated for 5 min and absorbance was measured at 593 nm in a 96-well plate in a microplate reader (Spectrostar Omega, BMG Ortenberg, Germany). The results were expressed as mmol of Fe Equivalents (FeE) 100 g^−1^ sample (d.m.).

### 2.8. Glycemic Index (GI)

The glycemic index (GI) was determined as described by Gularte and Rosell [38], with some modifications. Samples containing 50 mg of available starch were dissolved in 2 mL of Tris-maleate buffer (0.1 M, pH 6) and then 2 mL of enzyme solution containing porcine pancreatic amylase (460 U mL^−1^) and amyloglucosidase (6.6 U mL^−1^) were added. Aliquots of 150 μL were taken during the incubation period (120 min) and immediately the enzyme reaction was stopped in boiling water for 5 min and cooled on ice. Following this, a volume of 150 μL of absolute ethanol was added and the sample was centrifuged (10,000× *g*, 5 min). The pellet was washed with 200 μL of EtOH:H_2_O (1:1, *v*/*v*) and the supernatants were pooled. Subsequently, a GOPOD kit (Megazyme, Bray, Ireland) was used to perform colorimetric analysis of glucose. The values of the hydrolysis index (HI) and glycemic index (GI) were calculated with the formulas proposed by Grunfeld [39].

### 2.9. Determination of Anti-Inflammatory Activity (AIA)

Cell viability of murine RAW 264.7 macrophages (American Type Culture Collection, Manassas, VA, USA) was determined to address the cytotoxicity of the phenolic extracts. Stock solutions (10 mg/mL) of phenolic extracts were prepared in dimethyl sulfoxide and sterile filtered with a 0.22 μm polyvinylidene fluoride. Cells were grown in Dulbecco’s Modified Eagle Medium (DMEM, Life Technologies, Carlsbad, CA, USA) contained 10% (*v*/*v*) heat-inactivated fetal bovine serum (FBS, Life Technologies, Carlsbad, CA, USA) and 1% penicillin/streptomycin (Life Technologies, Carlsbad, CA, USA) at 37 °C with 5% CO_2_. Cell viability was determined using an MTS assay [23]. Briefly, cells were seeded in 96-well plates at a density of 5 × 10^4^ cells/well. After overnight attachment, cells were treated with 0.5 mg/mL of phenolic extracts diluted in growth medium, with the presence of 0.1 μg/mL of lipopolysaccharide from *Escherichia coli* O55:B5 (Sigma-Aldrich, St. Louis, MO, USA) for 24 h. After incubation, the cell culture media were collected for cytokine quantification and cells were treated with the Cell Titer 96 Aqueous One Solution Cell Proliferation Assay (Promega, Madison, WI, USA).

Cytokine analysis of the cell culture medium of macrophages was performed using the Mouse Cytokine Magnetic kit (Milliplex MCYTOMAG-70K-06, Merck Life Sciences, Madrid, Spain). This cytokine panel allows the simultaneous quantification of 5 mouse cytokines/chemokines, including MCP-1, IL-1β, IL-6, IL-10, INF-γ and TNF-α, based on fluorescence-encoded beads suitable for flow cytometry. A multiplex immunoassay was performed following the manufacturer’s recommendations. Data were acquired on a Luminex XYP flow cytometer (Luminex Co., Austin, TX, USA) and analyzed using the Belysa^TM^ Data Analysis Software (version 1.2). MCP-1 was over the detection limit whereas INF-γ was below the lower threshold in all the analyzed samples, thus they were excluded from the analysis.

### 2.10. Statistical Analysis

Data were expressed as the mean ± standard deviation of at least three independently performed experiments. Analysis of variance (ANOVA) and Duncan’s post hoc test were performed to detect differences between mean values. All statistical analyses, except quantification of phenolic compounds by HPLC-ESI-QTOF-MS, were performed with Statgraphics Centurion XVI^®^ (StatPoint Technologies, Inc., Warrenton, VA, USA).

The data of phenolics compounds quantified by HPLC-ESI-QTOF-MS were analyzed using IBM SPSS Statistics 28.0. Normality of the data was tested using the Shapiro–Wilk test. Due to the absence of normality, the Kruskal–Wallis test and Mann–Whitney’s U test, for comparisons between unrelated groups, were performed. Results are expressed as the mean values with their standard deviation. Significance was defined as a *p*-value < 0.05.

## 3. Results and Discussion

### 3.1. Proximal Composition

Proximal composition was evaluated in wheat grains and their corresponding brans (Table 1). The grains showed an ash content from 1.79 to 2.03 g 100 g^−1^, without significant differences between samples. Ash levels in brans were higher (*p* ≤ 0.05) than in grains, and values ranged between 6.26 to 7.16 g 100 g^−1^, the values being similar to the values reported by other authors [20] and slightly higher than the values reported by Chalamacharla et al. [40], who found values from 5.5 to 6.5 g 100 g^−1^ in WB.

Total dietary fiber (TDF) was significantly higher (*p* ≤ 0.05) in brans than in grains, as was expected, but without differences between grains or brans. TDF values in grains ranged from 14.41 to 15.46 g 100 g^−1^. In turn, in the brans, the values were in all the cases almost double (45.24 to 50.88 g 100 g^−1^). TDF is composed of soluble and insoluble dietary complex polysaccharides, such as cellulose, hemicelluloses and pentosan polymers linked to proteins and lignin [7]. As it has been previously reported by the authors, that more than 90% of wheat fiber is present as insoluble dietary fiber in grain (13.57 vs. 1.35 g 100 g^−1^ of insoluble and soluble fiber, respectively) and bran (52.37 vs. 1.55 g 100 g^−1^ of insoluble and soluble fiber, respectively) [20].

Dietary fiber increased satiety, which can be associated with the ability to absorb water, reducing the gut transit time, increasing the digesta viscosity in the small intestine and stool bulk, and increasing short-chain fatty acid production in the colon [41]. These physiological processes eventually lead to the health effects of dietary fiber, as mentioned above.

Fat, as it was expected, was very low in grains and brans, ranging between 1.73 and 1.90 g 100 g^−1^ in grains and from 3.87 to 4.17 g 100 g^−1^ in the brans. Similar fat content values were observed in grains; in brans, although not significantly (*p* ≥ 0.05), WB MP showed the highest values. 

Moisture content was significantly different (*p* ≤ 0.05) in brans compared to grains (Table 1); values oscillated between 12.58 and 12.62 g 100 g^−1^ in brans, with no significant differences between them. The moisture content in grains was lower than that in brans, with values from 9.28 to 10.34 g 100 g^−1^, and the lowest values within grains found in WG HP. The highest moisture observed in WG MP might respond to storing conditions; high moisture levels can be associated with a poor flow rate through the grain during its storage, which can affect to their quality and shelf life.

The milling factory provided grains with different protein levels. As expected, the protein content showed significant differences between the three grains. Protein contents of 10.75, 11.83 and 17.95 g 100 g^−1^ were found in WG LP, WG MP and WG HP, respectively. The protein values in brans ranged from 12.04 to 19.74 g 100 g^−1^. The wheat bran amino acids glutamic (18.6%) and aspartic acid (7.2%) are the most abundant amino acids according to the literature [42].

The results showed a higher phytic acid (PA) content in brans than in grains, with significant differences between samples, as was expected, since PA is most abundant in the pericarp and aleurone layer [43]. The phytic acid was higher in grain obtained from high protein grain (WG HP, Table 1). The levels in grains ranged from 0.70 to 0.83 g 100 g^−1^. These PA values were higher than reported by authors in previous studies, which can respond to agronomic practices, such as the reduction in fertilization in many cases. Deficiencies on fertilization have been associated with the content of phytic acid in the samples. On the other hand, PA values in brans ranged from 3.29 to 3.74 g 100 g^−1^, with the highest values observed in the sample with a high protein content (WB HP).

Phytic acid is one of the most important antinutrients in wheat, and a reduced bioavailability of certain minerals, such as iron, is partially associated with its presence. The mechanism is not clearly reported but it is suggested that phytic acid links with mineral cations to form complexes that modify mineral solubility and absorption [44]. However, other studies have associated beneficial properties to PA, such as a delayed post-prandial absorption [45], reduction in cholesterol and triglycerides [46] and anti-carcinogenic effects associated to their chelating properties [47].

Total starch content (TSC) was evaluated in all the samples, and the results showed a high variation regarding the type of ecotype analyzed. The TSC values in grains ranged from 48.30 g 100 g^−1^ to 54.37 g 100 g^−1^; meanwhile, in WB the highest values were low, ranging from 6.92 g 100 g^−1^ to 11.56 g 100 g^−1^. The differences observed may respond to the fact that most of the starch is located in the endosperm, which is composed of about 70% starch. WB obtained from medium-protein wheat showed a higher content of starch than that with a low- or high-protein content.

The TSC values observed were lower than those reported previously by other authors in grains and brans [20,48]. TSC variation could be attributed to variety, cultivation conditions and, in the specific case of brans, to the milling technique used for its separation, which determines the amount of starch attached to the aleurone layer after bran separation. Since the method employed in all the grains for bran production was the same, the differences observed should respond only to the differences associated to variety and agronomic practices.

### 3.2. Determination of Total Phenolic Compounds (TP)

**Figure 1:**
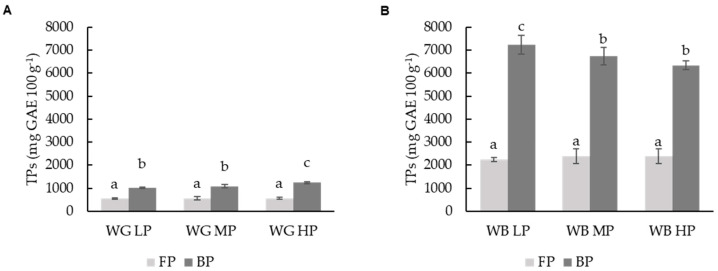
Total phenolic content (TP) of the free phenolic fraction (FP) and bound phenolic fraction (BP) of (**A**) different wheat grain (WG) and (**B**) wheat bran (WB) samples. Results are expressed in mg GAE 100 g^−1^ of d.m. Different letters indicate significant differences (*p* < 0.05). Abbreviations: WG LP: wheat grain low protein; WG MP: wheat grain medium protein and WG HP: wheat grain high protein, WB LP: wheat bran low protein; WB MP: wheat bran medium protein and WB HP: wheat bran high protein, d.m.: dry matter.

Total phenolic compounds were measured in wheat grains (WGs) and their respective wheat brans (WBs) as free and bound fractions, depending on the extraction method used (Figure 1). WBs exhibited a significantly (*p* ≤ 0.05) higher TP value than WGs, regardless of their protein content (high, medium and low) and the type of fraction (soluble or bound) studied.

The total phenol content corresponding to the free phenolic (FP) fraction were 5-fold times higher in brans than their correspondent grains, and 7-fold times in brans compared to grains regarding the bound phenolic (BP) fraction. TP ranged in grains from 538.38 to 561.80 mg GAE 100 g^−1^ in the FP fraction and from 1027.44 to 1236.07 mg GAE 100 g^−1^ in the BF fraction (Figure 1A). In brans, TP ranged from 1764 to 2547 mg GAE 100 g^−1^ and from 6344 to 7232 mg GAE 100 g^−1^ in FP and BP fractions, respectively. These results are in accordance with values previously reported by different authors in wheat [20,49].

Significant differences (*p* ≥ 0.05) were observed between free and bound fractions in grains; FP was not significantly affected by protein content (LP, MP, HP) in grain or bran. On the other hand, BP fractions were significantly (*p* ≥ 0.05) affected by protein content. Higher TP values were observed in the grain sample with the highest protein content (WG HP). In WB, an inverse correlation between protein and the TP of BP fraction was observed, with decreasing TP values corresponding to increasing protein content. The highest TP values in brans were observed in the WB LP, BP fraction. As explained, different trends were observed in relation to the TP and protein content in grain and bran. This could be due to different reasons. The bran is a tissue highly exposed to the environment, as compared to the inner parts of the grain, and it is there where a higher concentration of a certain type of phenolic compound, mostly associated with fiber, have been described. Ferulic acid was one of the main compounds identified in this study; WB LP is obtained from low-protein grains, which correspond to winter varieties. Yu and Beta, [50] reported higher levels in bound phenolic content in winter wheat varieties, as compared to spring varieties.

The inverse relationship between TP and protein content observed in the bran BP fractions, not observed in grains (Figure 1), could be associated with the interaction of phenolic compounds with proteins, which results in complexes that alter the structural properties of the proteins [51] and their solubility characteristics. Wheat endosperm contains mainly prolamins and glutelins, while albumins and globulins are most abundant in bran.

According to De Brier et al. [52], prolamins in wheat bran mainly originate from the endosperm; thus, the content depends on the amount of endosperm remaining in the bran fraction [53,54]. However, since the procedure used for obtaining all brans was identical, this effect could not explain the differences observed between WB HP, WB MP and WB LP. Nevertheless, Gammoh and colleagues [55] evaluated the antioxidant activity of the different protein fractions in wheat and found endosperm-related fractions (prolamins and glutelins) had a higher antioxidant capacity than bran-related proteins (albumins and globulins). These authors reported that the antioxidant activities of prolamins and glutelins were more affected by phenolic removal than albumins and globulins, suggesting prolamins and glutelins bind more phenolic compounds than albumins and globulins [56]. This may contribute to explain the higher positive correlation of protein and phenolic compounds in grains, where endosperm proteins are higher in proportion than in brans.

Globulins are one of the main proteins present in bran; in alkaline extracts of BP in bran, globulins have been reported to be associated to mainly vanillic acid, followed by chlorogenic acid, naringin and hesperidin. In acidic extracts, only hesperidin has been found [56]. Albumins, on the other hand, were found to bind the highest number of different phenolic compounds; the alkaline BP extract showed presence of identified phenolic compounds such as ferulic acid, chlorogenic acid, syringic acid, luteolin, quercetin and rutin. In the case of ferulic acid, this was the predominant individual phenolic compound bound to albumin [56].

### 3.3. Characterization of Phenolic Compounds by HPLC-ESI-QTOF-MS

**Figure 2:**
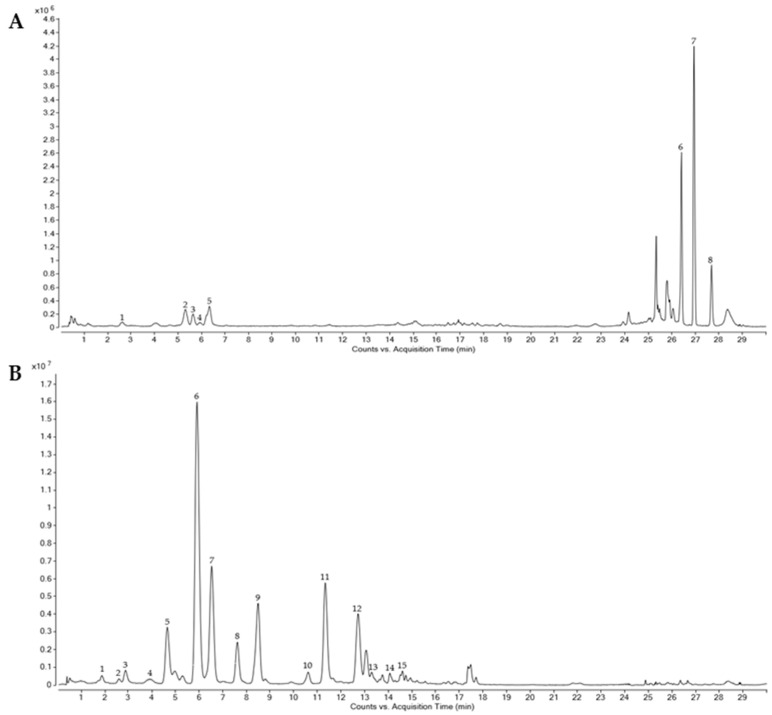
(**A**) Combined extracted ion chromatograms for the identified phenolic compounds in the free fraction of wheat: 1 = caffeic acid; 2 = 1-O-sinapoyl-beta-D-glucosa; 3 = apigenin-6-carabinoside-8-C-hexoside I; 4 = ferulic acid; 5 = apigenin-6-carabinoside-8-C-hexoside II; 6 = 5-nonadecenylresorcinol; 7 = 5-nonadecylresorcinol; 8 = 5-heneicosylresorcinol. (**B**) Combined extracted ion chromatograms for identified phenolic compounds in the bound fraction of wheat: 1 = hydroxybenzoic acid; 2 = caffeic acid; 3 = 4-hydroxybenzaldehyde; 4 = protocatechuic acid; 5 = p-coumaric acid; 6 = ferulic acid; 7 = isoferulic acid; 8 = diferulic isomer 1; 9 = diferulic isomer 2; 10 = diferulic isomer 3; 11 = diferulic isomer 4; 12 = diferulic isomer 5; 13 = diferulic isomer 6; 14 = syringaresinol; 15 = apigenin-6-C-galactosyl-8-C-glucosyl-O-glucuropyranoside.

HPLC-ESI-QTOF-MS was used to characterize the wheat grain and bran fractions with higher total phenol content (WG HP and WB MP), in order to elucidate the variation in their phenolic composition. Table 2 shows the twenty-two phenolic compounds that were identified in these wheat samples. The main compounds were classified as hydroxybenzoic acids (2), hydroxycinnamic acids (12), flavones (3), lignans (1), hydroxybenzaldehyde acids (1) and alkylphenols (3). Thus, a representative overall phenolic profile, based on selected ion extraction from the Total Ion Chromatogram, is depicted in Figure 2.

Phenolic compounds were confirmed based on their characteristic fragments described previously. Thus, the loss of a carbon dioxide ion (44 units) by collision-induced dissociation was observed in several of the identified compounds, such as ferulic acid, p-coumaric acid and caffeic acid. A loss of methyl radicals (15 units) was detected in ferulic acid and sinapic acid. This fragmentation pattern is similar to the reported collision-induced fragmentation of deprotonated methoxylated flavonoids in plant extracts [57] or of phenolic acids in different beverages [58]. Errors obtained in the samples were low, under ±10 ppm, confirming that these were the identified compounds (detailed information on the experimental and calculated *m*/*z* values for each compound and sample are provided as Table S1). Most phenolic compounds identified were present in all samples, WG being the sample with the lowest number of identified compounds (18).

The phenolic compounds quantified in the different wheat samples is presented in Table 3. The free phenolic (FP) fraction accounted for 3.1% and 8.7% of the total phenolic compounds in grain (WG) and bran (WB), respectively. The most abundant phenolic group in FP was alkylresorcinols, and particularly 5-nonadecylresorcinol reached 13.38 mg/100 g in WB. The total amount of alk(en)ylresorcinols was slightly over 20 mg/100 g, far from the levels found by other authors [59]. These compounds have an amphiphilic structure that provides them high bioaccessibility, as compared to other phenolic compounds [60], making these fractions (FP) of interest for bioactive ingredient and product development, despite its lower total phenolic concentration. A residual content of free phenolic acids was detected in the WB FP fraction (below 1.7 mg/100 g).

Regarding the distribution among the free and bound fractions, the number of phenolic compounds detected in the bound polyphenols fraction was higher than the observed in the free fraction. The bound phenolic (BP) fraction (associated with dietary fiber) ranged from 91.3% to 98.9%. It is well known that the high proportion of bound vs. free phenolic compounds in cereals is due to their association with the dietary fiber fraction [61], exerting synergistic interactions that result in health benefits such as reduced risk of chronic diseases [62]. Ferulic acid (FA) was the major component in BP (61.33 and 177.49 mg/100 g in WG and WB, respectively). FA combined with ferulic dimers and isoferulic acid represented 96.5% of the phenolic compounds in WB, and 94.6% in WG. These results are similar to those reported previously in other studies, showing that FA and its dimers are ubiquitously found in cereal varieties and fractions, and are particularly abundant in the bound fraction [20,63].

Only two phenolic compounds were identified in both the FP and BP fractions: ferulic acid and caffeic acid. These results agreed with the results observed in previous works [10,12,23].

Regarding comparison between whole grain and bran, the WB total phenolic compound content, calculated as the sum of FP and BP, in WB was three-fold higher than that in WG (466.8 ± 5.06 mg/100 g vs. 123.53 ± 9.31 mg/100 g). This has been reported in other studies, where the bran showed higher amounts of phenolic compounds than the whole grain, although the exact amount depends upon the variety and milling process [61,64].

### 3.4. Total Antioxidant Capacity (TAC)

The total antioxidant capacity (TAC) of the samples was evaluated using four methods: 2,2-diphenyl- l -picrylhydrazyl assay (DPPH assay), 2,2′-azino-bis (3-ethylbenzothiazoline-6-sulfonic acid) (ABTS^•+)^, ferric reducing ability assay (FRAP) and oxygen radical absorbance capacity (ORAC).

**Figure 3:**
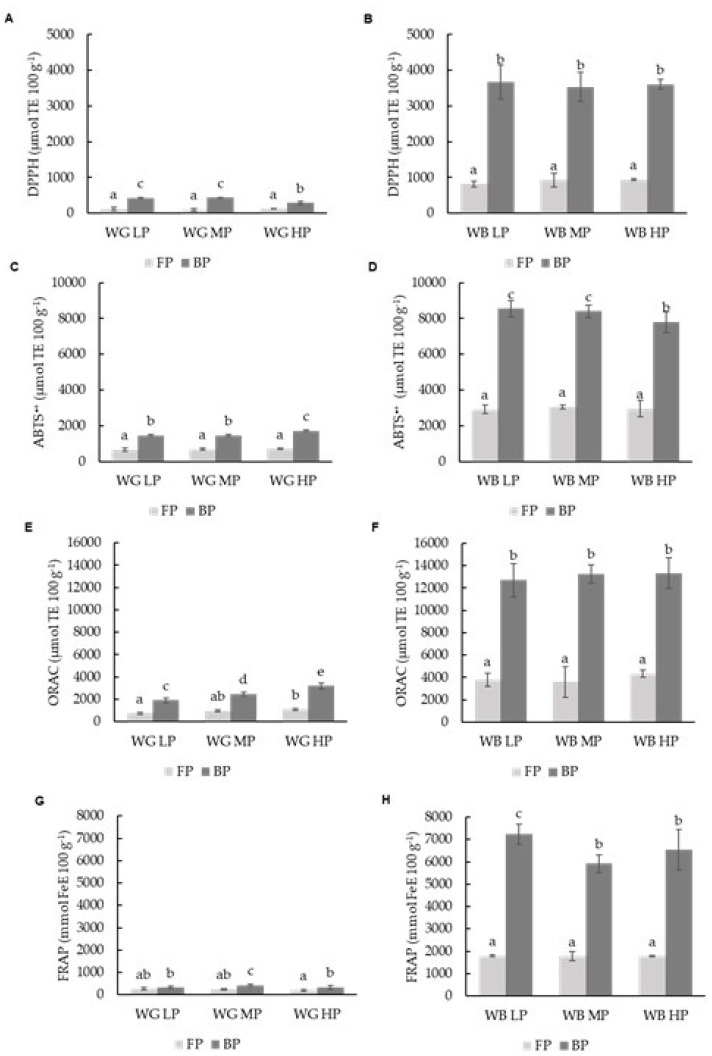
DPPH (**A**,**B**), ABTS^•+^ (**C**,**D**), ORAC (**E**,**F**) and FRAP (**G**,**H**) values for free phenolic fraction (FP) and bound phenolic fraction (BP) of different wheat grain (WG) and wheat bran (WB) samples. Results were expressed in μmol TE 100 g^−1^ of d.m for DPPH, ABTS•+, ORAC and Fe E 100 g^−1^ of d.m for FRAP. Different letters indicate significant differences (*p* < 0.05). Abbreviations: WG LP: wheat grain low protein; WG MP: wheat grain medium protein and WG HP: wheat grain high protein, WB LP: wheat bran low protein; WB MP: wheat bran medium protein and WB HP: wheat bran high protein, d.m.: dry matter.

Free phenolic (FP) values were 8-fold times lower in grains, as compared to those of the brans, ranging from 98.36 to 120.61 µmol eq. Trolox 100 g^−1^, and bound phenolics (BP) were 12-fold times reduced, ranging from 288.50 to 435.21 µmol eq. Trolox 100 g^−1^ (Figure 3A). Meanwhile, it was observed that the DPPH radical scavenging capacity of the brans of the three wheat varieties studied (WB HP, WB MP and WB LP) was higher than that of their respective grains (Figure 3B). Values ranged from 819.49 to 941.16 µmol eq. Trolox 100 g^−1^ in FP fractions and from 3667.01–3536.47 µmol eq. Trolox 100 g^−1^ in the BP fractions in bran.

Significant (*p* ≤ 0.05) correlations between total phenols and DPPH were observed in the FP and BP fractions of grains and brans. These antioxidant assays evaluated the capacity of the antioxidants for both electron and hydrogen atom transfer reactions for radical scavenging [65,66], and although not a natural radical, the DPPH assay offers a mechanism of reaction similar to those occurring in the neutralization of peroxyl radicals [67]. In this sense, phenolic compounds present in the grain and bran extracts would effectively prevent peroxyl-mediated reactions, such as lipid oxidation reactions [68].

Most of the phenolic compounds are located in the wheat bran layer covalently cross-linked with the cell wall polymers [49,69]; the phenolic content ratio in bran, as compared to germ, is 15- to 18-fold, as has been reported previously, and in agreement with different authors, this correspond mostly to phenolic acids [70]. No significant differences due to protein content were observed in the DPPH results, with exception of a lower DPPH activity in the WG HP bound phenolic (BP) fraction, as compared to the other two BP fractions with lower protein (WG LP and WG MP) in the grains. Other authors have found a significant correlation between the antioxidant activity and total phenolic content in the albumin protein fraction in wheat [56]. The decrease in DPPH scavenging capacity of the WG HP-BP fraction, in relation to the low- and medium-protein grains (WG LP and WG MP), may be due to alteration in the mechanism of reaction in the DPPH method due to changes in phenolic concentration, as has been suggested previously [71].

The different fractions of grains and brans were also evaluated for their antioxidant activity with an ABTS^•+^ assay. This method, similar to DPPH, evaluates both the electron and hydrogen atom transfer reactions of antioxidants [65,66]. Bran showed significantly higher antioxidant activity than grain, where the antioxidant activity was 4-fold higher in the free and bound fraction (Figure 3C,D). Contrary to the results observed in the DPPH grains, the high-protein variety (WG HP) showed higher ABTS•+•+-scavenging capacity than varieties with a lower protein content (WG LP, WG MP); this would be in line with previous results, where a significant correlation between the prolamin protein fractions (mostly present in endosperm) and the antioxidant activity of phenolic compounds has been reported, linked to their antioxidant capacity [56].

The ORAC method (oxygen radical absorption capacity) was also used to assess the antiradical capacity of the different fractions, as estimation of the grain and bran peroxyl scavenging capacity through hydrogen atom transfer reactions [72]. As expected from the phenolic compound results, the bran FP and BP fractions were significantly higher than the grain FP and BP fractions (Figure 3E,F); the antioxidant activities of FP and BP were 4-fold and 5-fold higher, respectively, in bran than in grain. The ORAC results showed a good correlation and similar trend than the phenolic compound values, although no significant reduction in ORAC activity was observed with increasing protein content in the case of the BP fractions in bran, as was the case with the phenolic content results.

The reducing power (FRAP assay) was evaluated in all grains and brans, the FP and BP fractions. The differences observed in the FRAP results for WG, as compared with WB, were much lower than those obtained from the total phenolic and antioxidant assays, and were in the range of 10- to 20-fold lower (Figure 3G,H). This may be due to the different profiles observed in the phenolic compounds identified (Table 3). As previously reported, alkyl derivatives, such as the alkylresorcinols found in the FP fractions, show lower reducing capacities than phenolic acids [73], such as ferulic acid, which was not present in the FP fractions, or to a very limited extent.

### 3.5. Glycemic Index (GI)

**Figure 4:**
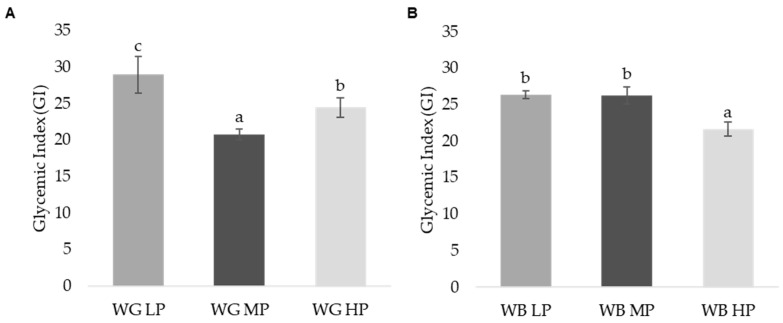
Glycemic index (GI) values for different (**A**) wheat grain (WG) and (**B**) wheat bran (WB) samples. Different letters indicate significant differences (*p* < 0.05). Abbreviations: WG LP: wheat grain low protein; WG MP: wheat grain medium protein and WG HP: wheat grain high protein, WB LP: wheat bran low protein; WB MP: wheat bran medium protein and WB HP: wheat bran high protein, FP: free phenolic fraction, BP: bound phenolic fraction, d.m.: dry matter.

The glycemic index (GI) was estimated in vitro in the WG and WB samples as a simplified approach to potential health-related properties and improved glycemic control of these matrices. Results showed significantly (*p* ≤ 0.05) lower GI values (20–30) than the white bread reference (100) (Figure 4); this significant difference can be related to the structure of non-gelatinized starch, as the rate of hydrolysis increases proportionally with the degree of starch gelatinization [74]. In all the cases, the values were lower than 30, which would indicate a low GI. The general trend observed was that the increasing protein content of the grain or bran results in a lower glycemic index. Different protein content can determine tighter or less tight interaction between the starch matrix and gluten. Bran samples showed lower GI than their corresponding grain sample, with the exception of MP wheat. This may be explained by differences in phytic acid concentrations (Table 1). Phytic acid inhibits α-amylase activity through interactions with divalent cations [45]. It has been shown that breads prepared with different phytate concentrations significantly reduced their in vitro starch digestibility and GI [45].

**Figure 5:**
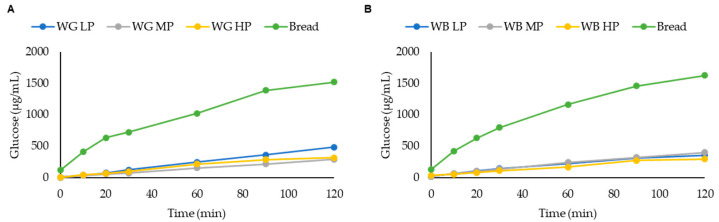
Glucose kinetics consumption (μg mL^−1^) for different (**A**) wheat grain (WG) and (**B**) wheat bran (WB) samples. Different letters indicate significant differences (*p* < 0.05). Abbreviations: WG LP: wheat grain low protein; WG MP: wheat grain medium protein and WG HP: wheat grain high protein, WB LP: wheat bran low protein; WB MP: wheat bran medium protein and WB HP: wheat bran high protein.

The hydrolysis curves of the grains and brans are shown in Figure 5, and the samples were compared with white bread as reference. No significant differences were observed between samples in their kinetic digestion up until 90 min, where WG MP and WG HP showed lower glucose production as compared to WG LP and bread. In the case of WB samples, the sample with the highest protein content (WB HP) resulted in the lowest GI value. The in vitro assessment of GI does not fully reflect the physiological response of the organism (hormone-mediated regulation of glycemia). On the other side, individual variability is avoided, and this assay shows the glycemic index as an inherent property of the starch and its interactions with other macromolecules. Since no significant differences in TDF were reported between the WG or WB samples (Table 1), the protein content may be responsible for the observed differences. It has been previously described as a potential mechanism for protein-mediated GI reduction, the role of the protein structure in protecting starch from gelatinization [75], or the presence of antinutrients, such as phytic acid, which may bind to proteins closely associated to starch, in synergy with other factors such as chelation of Ca required for α-amylase activity [76].

### 3.6. Anti-Inflammatory Activity

**Figure 6:**
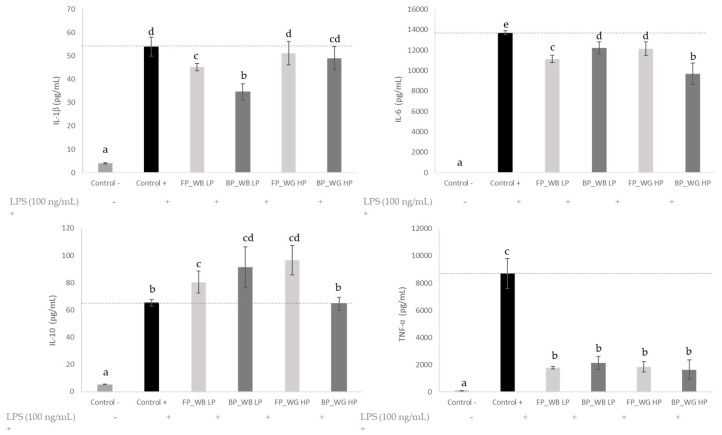
Macrophages cytokine milieu of culture supernatants treated with phenolic extracts obtained from WG HP and WB LP (0.5 mg mL^−1^). Macrophages were cultured in a medium (control −), 100 ng mL^−1^ LPS (control +) or 100 ng mL^−1^ LPS + free (FP) and bound (BP) phenolic fractions, for 24 h. Data are the means ± standard deviation (*n* = 4). Different letters denote statistical dif-ferences among the experimental groups. Abbreviations: LPS: lipopolysaccharide from Esche-richia coli; WG HP: wheat grain high protein; WB LP: wheat bran low protein.

The regulatory role of the immune response is essential to maintain tissue homeostasis in all organs and systems of the body. Unfortunately, when this mechanism is dysregulated, an exacerbated immune response may lead to chronic inflammation and represent the underlying pathological cause of many disorders. Therefore, we determined if FP and BP extracts from WG HP and WB LP displayed modulatory effects on the protein levels of several immune mediators in murine macrophages in the presence of a pro-inflammatory insult, such as the Gram-negative bacteria endotoxin LPS (Figure 6). Referred to the resting cultures (control -), LPS (control +) significantly induced the secretion of pro-inflammatory IL-1β, IL-6, IL-10 and TNF-α (*p* < 0.05). Noteworthy, the phenolic extracts obtained from 0.5 mg/mL of WB and WG had the capacity to revert the LPS-induced proinflammatory effects. Overall, the WB LP phenolic extracts modestly inhibited IL-1β, IL-6 secretion, and strongly the TNF-α levels (*p* < 0.05), whereas they stimulate IL-10 secretion (*p* < 0.05)—an anti-inflammatory cytokine able to inhibit the synthesis of proinflammatory cytokines in macrophages. Our results are in line with previous studies providing evidence of the anti-inflammatory effects of wheat phenolic compounds in LPS-induced RAW 264.7 macrophages [22,23,48]. Particularly, WB polyphenols have demonstrated modulatory actions, inhibiting the production of pro-inflammatory cytokines TNF-α and IL-6 in LPS-induced RAW264.7 macrophages. The mechanisms underlying the effect in this cell line could involve modulation of cycloxygenase-2/prostaglandin E2, inducible nitric oxide synthase/nitric oxide and nuclear factor (NF)-𝜅B pathways, as has been reported in the human colonic adenocarcinoma cell (HT-29) line under a TNFα inflammatory insult [77].

The anti-inflammatory effect of WB LP was more evident for the BP fraction, showing a stronger inhibition of IL-1β and enhancement of IL-10 secretion as compared to FP. This finding could be attributed to the high abundance and diversity of phenolic compounds in the BP fraction (Table 3). Ferulic acid and its derivatives, a major phenolic compound in BP extracts of WB, prevented inflammation and oxidative stress in LPS-stimulated RAW264.7 cells by suppression of intracellular ROS and inflammatory mediators, including iNOS, COX-2, TNF-alpha and IL-6 [78]. Besides, in a recent in vivo study of El-gogary et al. [79], the administration of FA in lipid nanocapsules reduced significantly the TNF-α and NF-kB levels in colorectal cancer-induced rats. The anti-inflammatory effect of WG HP was also confirmed in the present study although a weaker anti-inflammatory effect was observed with respect to WB LP. Specifically, the FP and BP extracts from WG HP did not significantly inhibit IL-1β or enhanced IL-10 secretion (in the case of BP), respectively (*p* > 0.05). This weaker effect could be explained by the lower abundance of phenolic compounds in WG HP phenolic extracts.

## 4. Conclusions

A significantly higher content of phenolic compounds was found in bran fractions, both free and bound, as compared to the whole grain fractions—differences that ranged from 4- to 6-fold. Similar results have been reported previously for wheat, and this pattern can be observed also in related species of ancient wheats. To some extent, an effect of the protein content over the resulting phenolic content and antioxidant capacity was observed, especially in grain, but also in bran, although in the latter a negative correlation was observed, and an increasing protein content resulted in decreasing total phenolic content, antioxidants and ferric reducing capacities.

A significant effect of the protein content on the GI of the WG and WB samples was also found, which may be explained by the structural effect of proteins associated with starch, reducing its gelatinization kinetics, or facilitating antinutrient–protein interactions that can reduce the GI.

The presence of phenolic compounds affects the various biological properties of the wheat protein fractions, including the antioxidants and anti-inflammatory activity. These effects arise through interactions between the compounds and protein that lead to structural and conformational changes. RP-HPLC analysis revealed that for protein isolates from white wheat flour, the phenolic compounds differed between the free phenolic group and bound phenolic.

The authors highlighted the better capacity of WB LP, specially the BP fraction, to inhibit the production of pro-inflammatory cytokines and expanding the production of anti-inflammatory IL-10 in macrophages in the presence of LPS. These results provide new insights into developing functional products with potential immunomodulatory activities to prevent chronic diseases through the valorisation of WB as a source of bioactive compounds. Green processes to release WB BP need to be further explored and offer clues to design effective functional ingredients.

## Figures and Tables

**Table 1:** Proximal composition of different wheat grain (WG) and wheat bran (WB) samples. Values were expressed as g 100 g^−1^ of dry matter. Different letters in the same row indicate significant differences (*p* < 0.05). Abbreviations: WG LP: wheat grain low protein; WG MP: wheat grain medium protein and WG HP: wheat grain high protein, WB LP: wheat bran low protein; WB MP: wheat bran medium protein and WB HP: wheat bran high protein, TDF: total dietary fibre; PA: phytic acid; TSC: total starch content), d.m: dry matter.

	WG LP	WG MP	WG HP	WB LP	WB MP	WB HP
**Ash**	1.79 ± 0.09 ^a^	1.81 ± 0.19 ^ab^	2.03 ± 0.23 ^b^	6.32 ± 0.17 ^c^	6.26 ± 0.08 ^c^	7.16 ± 0.30 ^d^
**TDF**	15.46 ± 0.91 ^a^	14.41 ± 0.79 ^a^	14.64 ± 0.06 ^a^	50.88 ± 19.90 ^b^	45.24 ± 13.93 ^b^	46.04 ± 17.86^b^
**Fat**	1.73 ± 0.01 ^a^	1.90 ± 0.40 ^a^	1.80 ± 0.33 ^a^	3.87 ± 0.06 ^b^	3.86 ± 0.20 ^b^	4.17 ± 0.28 ^b^
**Moisture**	10.13 ± 0.13^b^	10.34 ± 0.16 ^b^	9.28 ± 0.18 ^a^	12.59 ± 0.93 ^c^	12.58 ± 0.61 ^c^	12.62 ± 1.04 ^c^
**Proteins**	10.75 ± 0.46 ^a^	11.82 ± 0.56 ^b^	17.95 ± 0.57 ^d^	12.04 ± 0.58 ^b^	15.16 ± 0.40 ^c^	19.31 ± 0.28 ^e^
**Carbohydrates**	85.73 ± 0.38 ^f^	84.47 ± 0.03 ^e^	78.21 ± 0.01 ^d^	77.73 ± 0.35 ^c^	74.73 ± 0.12 ^b^	69.36 ± 0.30 ^a^
**PA**	0.75 ± 0.00 ^b^	0.70 ± 0.01 ^a^	0.83 ± 0.02 ^c^	3.29 ± 0.03 ^d^	3.55 ± 0.02 ^e^	3.74 ± 0.00 ^f^
**TSC**	50.24 ± 2.00 ^d^	48.30 ± 1.69 ^c^	54.37 ± 1.22 ^e^	8.77 ± 0.62 ^a^	11.56 ± 0.83 ^b^	6.92 ± 0.30 ^a^

**Table 2:** Phenolic compounds identified in WG and WB samples by HPLC-ESI-QTOF-MS. Abbreviations: WG: wheat grain, WB: wheat bran, FP: free phenolic fraction, BP: bound phenolic fraction.

Class	Sub-Class	Compound	Molecular Formula	Ion Fragments	WB		WG	
					Error	Fraction	Error	Fraction
Phenolic acids	Hydroxybenzoic acids	Protocatechuic acid	C_7_H_6_O_4_	151. 136. 112	−7.58	BP	−4.34	BP
		Hydroxybenzoic acid	C_7_H_6_O_3_	135. 125. 121	−7.12	BP	−4.22	BP
	Hydroxycinnamic acids	Ferulic acid	C_10_H_10_O_4_	178. 149. 134	−3.96	FP-BP	0.17	BP
		p-Coumaric acid	C_9_H_8_O_3_	119	8.95	BP	n.d.	n.d.
		Sinapic acid	C_11_H_12_O_5_	208	n.d.	n.d.	−1.8	BP
		Diferulic isomer 1	C_20_H_18_O_8_	341. (282). 193. (112)	−1.84	BP	−2.1	BP
		Diferulic isomer 2	C_20_H_18_O_8_	359. 341. 326	−1.58	BP	−2.35	BP
		Diferulic isomer 3	C_20_H_18_O_8_	369. 355. 341. 313. 271	−3.39	BP	1.01	BP
		Diferulic isomer 4	C_20_H_18_O_8_	341. 326. 282. 248. 227	4.38	BP	−2.87	BP
		Diferulic isomer 5	C_20_H_18_O_8_	347. 313. 261. 217. 193. 178	0.49	BP	−2.35	BP
		Diferulic isomer 6	C_20_H_18_O_8_	341. 303. 239. 193. 178	−1.32	BP	n.d.	n.d.
		Caffeic acid	C_9_H_8_O_4_	(165). 135. 127	−2.32	FP-BP	−5.65	BP
		Isoferulic acid	C_10_H_10_O_4_	178. 149. 134	4.29	BP	−0.86	BP
		1-O-Sinapoyl-beta-D-glucose	C_17_H_22_O_10_	216. 162. 119	−4.87	FP	−2.54	FP
Flavonoids	Flavones	Apigenin-6-C-arabinoside-8-C-hexoside I	C_26_H_28_O_14_	397. 316. 216. 119	1.82	FP	0.94	FP
		Apigenin-6-C-arabinoside-8-C-hexoside II	C_26_H_28_O_14_	432. 245. 164. 149	−1.37	FP	−1.01	FP
		Apigenin-6-C-galactosyl-8-C-glucosyl-O-glucuropyranoside	C_33_H_38_O_21_	577. 343	5.3	BP	n.d.	n.d.
Lignans	Lignans	Syringaresinol	C_22_H_26_O_8_	387. 353. 341. 257. 193. 119	n.d.	n.d.	−1.93	B
Others	Hydroxybenzaldehyde acids	4-Hydroxybenzaldehyde	C_7_H_6_O_2_	(112)	−4.89	BP	−4.07	B
	Alkylphenols	5-Nonadecenylresorcinol	C_25_H_42_O_2_	347. 309. 283	−6.67	FP	n.d.	n.d.
		5-Nonadecylresorcinol	C_25_H_44_O_2_	(355. 337. 311. 279. 248)	−4.37	FP	−3.04	FP
		5-Heneicosylresorcinol	C_27_H_48_O_2_	(379). 339. 248	−2.83	FP	−4.32	FP

**Table 3:** Phenolic compounds (mg 100 g^−1^ d.m.) quantified in wheat samples by HPLC-ESI-QTOF-MS. Different letters in the same row indicate significant differences (*p* < 0.05). Abbreviations: WG: wheat grain; WB: wheat bran, FP: free phenolic fraction, BP: bound phenolic fraction; n.d.: not detected; LOD: limit of detection.

Class	Sub-Class	Compound	OG		OB		SO		OH1	OH2
			FP	BP	FP	BP	FP	BP		
Phenolic acids	Hydroxybenzoic acids	Protocatechuic acid	1.05 ± 0.04 ^a^	n.d.	n.d.	n.d.	n.d.	1.71 ± 0.17 ^b^	n.d.	n.d.
		Hydroxybenzoic acid	n.d.	0.75 ± 0.01 ^c^	0.53 ± 0.02 ^a^	n.d.	n.d.	0.66 ± 0.10 ^b^	n.d.	0.74 ^c^
	Hydroxycinnamic acids	Ferulic acid	0.36 + 0.01	28.30 ± 1.56 ^c^	0.50 + 0.13	213.76 ± 4.39 ^e^	n.d.	32.36 ± 3.09 ^d^	6.15 ± 0.87^b^	3.38 ^a^
		p-Coumaric acid	n.d.	n.d.	n.d.	130.67 ± 1.43 ^c^	n.d.	n.d.	11.47 ± 2.11 ^b^	7.32 ^a^
		Sinapic acid	n.d.	2.93 ± 0.18 ^b^	n.d.	n.d.	n.d.	2.50 ± 0.14 ^a^	n.d.	n.d.
		Avenanthramide C	n.d.	n.d.	0.21 ± 0.02 ^a^	n.d.	13.40 ± 0.34 ^b^	0.19 ± 0.04 ^a^	n.d.	n.d.
		Avenanthramide 2p	n.d.	n.d.	1.58 ± 0.14 ^a^	n.d.	14.35 ± 0.27 ^b^	n.d.	n.d.	n.d.
		Avenanthramide 2f	0.23 ± 0.07 ^a^	n.d.	1.48 ± 0.10 ^b^	n.d.	19.24 ± 0.19^d^	1.67 ± 0.01 ^c^	n.d.	n.d.
		Diferulic isomer 1	n.d.	4.42 ± 0.22 ^b^	n.d.	n.d.	n.d.	3.80 ± 0.38 ^a^	n.d.	n.d.
		Diferulic isomer 2	n.d.	5.02 ± 0.63 ^b^	n.d.	n.d.	n.d.	3.85 ± 0.38 ^a^	n.d.	n.d.
		Diferulic isomer 4	n.d.	5.70 ± 0.39 ^a^	n.d.	n.d.	n.d.	8.35 ± 0.82 ^b^	n.d.	n.d.
		Diferulic isomer 5	n.d.	6.83 ± 0.59 ^a^	n.d.	n.d.	n.d.	7.30 ± 0.62 ^a^	n.d.	n.d.
		Diferulic isomer 6	n.d.	0.74 ± 0.06 ^a^	n.d.	n.d.	n.d.	n.d.	n.d.	n.d.
		Caffeic acid	2.32 ± 0.01 ^c^	1.50 ± 0.11 ^a^	n.d.	6.42 ± 0.26 ^e^	n.d.	3.44 ± 0.41 ^d^	2.41 ± 0.25 ^c^	1.72 ^b^
		Isoferulic acid	n.d.	3.36 ± 0.67 ^a^	n.d.	n.d.	n.d.	2.96 ± 0.39 ^a^	n.d.	n.d.
		1-O-Sinapoyl-beta-D-glucose	1.55 ± 0.02 ^a^	n.d.	n.d.	n.d.	2.45 ± 0.02 ^b^	n.d.	n.d.	n.d.
Flavonoids	Flavones	Apigenin-6-C-arabinoside-8-C-hexoside III	n.d.	n.d.	<LOD	n.d.	<LOD	n.d.	n.d.	n.d.
Others	Hydroxybenzaldehide acids	4-Hydroxybenzaldehyde	n.d.	1.10 ± 0.11 ^a^	4.17 ± 0.06 ^c^	27.88 ± 1.44 ^e^	n.d.	1.71 ± 0.11 ^b^	9.10 ± 1.05 ^d^	9.91 ^d^

## Supplementary Materials

The following supporting information can be downloaded at: https://www.mdpi.com/article/10.3390/foods11142049/s1. Table S1. m/z values of phenolic compounds obtained in wheat samples by HPLC-ESI-QTOF-MS.
