# Supplementary material for: Impact of Protein Content on the Antioxidants, Anti-Inflammatory Properties and Glycemic Index of Wheat and Wheat Bran
Source: Foods. 2022 Jul 11;11(14):2049. doi: 10.3390/foods11142049 (PMC9322734; doi:10.3390/foods11142049)
Supplement: Supplementary file 1 [file foods-11-02049-s001.zip › foods-1794119-Supplementary.pdf]

Table S1. *m*/*z* values of phenolic compounds obtained in wheat samples by HPLC-ESI-QTOF-MS. Abbreviation: WB = wheat bran, WG = wheat grain, n.d. = not detected.

**Compound**	**WB**		**WG**	
	***m*/*z* exp**	***m*/*z* calc**	***m*/*z* exp**	***m*/*z* calc**
Protocatechuic acid	153.0205	153.0193	153.0200	153.0193
Hydroxybenzoic acid	137.0254	137.0244	137.0250	137.0244
Ferulic acid	193.0514	193.0506	193.0506	193.0506
p-Coumaric acid	163.0386	163.0401	n.d.	n.d.
Sinapic acid	n.d.	n.d.	223.0616	223.0612
Diferulic isomer 1	385.0636	385.0929	385.0937	385.0929
Diferulic isomer 2	385.0935	385.0929	385.0938	385.0929
Diferulic isomer 3	385.0942	385.0929	385.0925	385.0929
Diferulic isomer 4	385.0912	385.0929	385.0940	385.0929
Diferulic isomer 5	385.0927	385.0929	385.0938	385.0929
Diferulic isomer 6	385.0934	385.0929	n.d.	n.d.
Caffeic acid	179.0354	179.0350	179.0360	179.0350
Isoferulic acid	193.0498	193.0506	193.0508	193.0506
1-O-Sinapoyl-beta-D-glucose	385.1159	385.114	385.1150	385.114
Apigenin-6-C-arabinoside-8-C-hexoside I	563.1396	563.1406	563.1401	563.1406
Apigenin-6-C-arabinoside-8-C-hexoside II	563.1414	563.1406	563.1412	563.1406
Apigenin-6-C-galactosyl-8-C-glucosyl-O-glucuropyranoside	769.1792	769.1833	n.d.	n.d.
Syringaresinol	n.d.	n.d.	417.1563	417.1555
4-Hydroxybenzaldehyde	121.0301	121.0295	121.0300	121.0295
5-Nonadecenylresorcinol	373.3137	373.3112	n.d.	n.d.
5-Nonadecylresorcinol	375.3285	375.3269	375.3280	375.3269
5-Heneicosylresorcinol	403.3593	403.3582	403.3599	403.3582
